# Long-Term Protection of CHBP Against Combinational Renal Injury Induced by Both Ischemia–Reperfusion and Cyclosporine A in Mice

**DOI:** 10.3389/fimmu.2021.697751

**Published:** 2021-07-26

**Authors:** Yufang Zhang, Yuanyuan Wu, Wei Wang, Feng Liu, Yiwen Zhang, Cheng Yang, Aifen Liu, Jing Wu, Tongyu Zhu, Michael L. Nicholson, Yaping Fan, Bin Yang

**Affiliations:** ^1^ Renal Group, Basic Medical Research Centre, Medical College of Nantong University, Nantong, China; ^2^ Nantong-Leicester Joint Institute of Kidney Science, Department of Nephrology, Affiliated Hospital of Nantong University, Nantong, China; ^3^ Department of Urology, Zhongshan Hospital, Shanghai Key Laboratory of Organ Transplantation, Fudan University, Shanghai, China; ^4^ Department of Cardiovascular Sciences, University of Leicester, University Hospitals of Leicester, Leicester, United Kingdom; ^5^ Department of Surgery, Addenbrooke’s Hospital, University of Cambridge, Cambridge, United Kingdom

**Keywords:** apoptosis, CASP-3, cyclic helix B peptide, cyclosporine A, ischemia–reperfusion injury

## Abstract

Renal ischemia–reperfusion (IR) injury and cyclosporine A (CsA) nephrotoxicity affect allograft function and survival. The prolonged effects and underlying mechanisms of erythropoietin derived cyclic helix B peptide (CHBP) and/or caspase-3 small interfering RNA (CASP-3siRNA) were investigated in mouse kidneys, as well as kidney epithelial cells (TCMK-1), subjected to transplant-related injuries. Bilateral renal pedicles were clamped for 30 min followed by reperfusion for 2 and 8 weeks, with/without 35 mg/kg CsA gavage daily and/or 24 nmol/kg CHBP intraperitoneal injection every 3 days. The ratio of urinary albumin to creatinine was raised by IR injury, further increased by CsA and lowered by CHBP at 2, 4, 6 and 8 weeks, whereas the level of SCr was not significantly affected. Similar change trends were revealed in tubulointerstitial damage and fibrosis, HMGB1 and active CASP-3 protein. Increased apoptotic cells in IR kidneys were decreased by CsA and CHBP at 2 and/or 8 weeks. p70 S6 kinase and mTOR were reduced by CsA with/without CHBP at 2 weeks, so were S6 ribosomal protein and GSK-3β at 8 weeks, with reduced CASP-3 at both time points. CASP-3 was further decreased by CHBP in IR or IR + CsA kidneys at 2 or 8 weeks. Furthermore, in TCMK-1 cells CsA induced apoptosis was decreased by CHBP and/or CASP-3siRNA treatment. Taken together, CHBP predominantly protects kidneys against IR injury at 2 weeks and/or CsA nephrotoxicity at 8 weeks, with different underlying mechanisms. Urinary albumin/creatinine is a good biomarker in monitoring the progression of transplant-related injuries. CsA divergently affects apoptosis in kidneys and cultured kidney epithelial cells, in which CHBP and/or CASP-3siRNA reduces inflammation and apoptosis.

## Introduction

Kidney transplantation is the life-changing treatment for patients with end-stage renal disease, whereas chronic allograft dysfunction (CAD) and donor shortage are still major obstacles (1). Ischemia–reperfusion (IR) injury, unavoidable in organ transplantation, is associated with delayed graft function, acute rejection, and subsequent CAD (2). IR injury initiates immune responses, oxidative damage, inflammation and cell death, in which tubular epithelial cells (TECs) are most vulnerable (3–5). Survived TECs participate in alleviating injury and promoting repair (6) *via* dedifferentiating and entering cell cycle within a few hours post injury (7). Cyclosporine A (CsA) as an immunosuppressant was used after kidney transplantation to reduce acute rejection and early graft losses (8). However, CsA has not improved long-term graft survival due to its nephrotoxicity (9), characterized by tubulointerstitial fibrosis and afferent arteriolar hyalinosis (10).

Erythropoietin (EPO) protected solid organs including kidneys against IR injury through a heterodimer EPO receptor and β-common receptor (EPOR/βcR), also named as innate repair receptor. EPOR/βcR is pharmacologically distinct from the homodimer receptor of (EPOR)_2_, a mediator of erythropoiesis (11–13). We showed that EPO protected kidneys against IR injury by decreasing tubular cell apoptosis, but promoting inflammatory cell apoptosis (4, 14, 15). However, the renoprotection of EPO required a large dosage that often causes hypertension and thrombosis (16). Helix B surface peptide (HBSP) was then developed from the 3D structure of EPO, composed of 11 amino acids. HBSP only interacts with EPOR/βcR, without stimulating (EPOR)_2_, but with a short serum half-life only few minutes (17). A novel metabolic stable cyclic HBSP (CHBP) was further produced by Chinese scientists, with prolonged half-life and potent renoprotection (17–20).

Caspase-3 (CASP-3) is a crucial player in CAD, which executes apoptosis and inflammation in transplant-related renal injuries (21, 22). In addition, HMGB1, a damage-associated molecule, is rapidly released from nuclei to extracellular domains upon injury, mediating apoptosis and inflammation (23), and subsequent fibrosis (24, 25). Our previous study showed that CASP-3 and HMGB1 were associated with the degree of renal IR injury and fibrosis (22, 26), while CHBP and CASP-3 siRNA (CASP-3siRNA) reduced CASP-3 and HMGB1 and improved renal injury (18, 22). CHBP improved endoplasmic reticulum stress at 12 h, inhibited inflammation and apoptosis at 5 and 7 days in mouse IR kidneys (18, 26), and also effectively restored kidney function and structure in isolated porcine IR kidneys and kidney grafts with acute rejection (19, 27, 28). In addition, the continuous administration of HBSP mitigated CASP-3, apoptosis, inflammation and fibrosis in 2-week IR ± CsA kidneys, while CHBP administered at the onset of injury reduced renal fibrosis after 12-week IR (19, 20). CHBP protected aristolochic acid induced acute kidney injury and unilateral ureter obstruction induced tubulointerstitial fibrosis (29, 30). CHBP also ameliorated acute myocardial infarction and acute lung injury by inhibiting apoptosis and inflammatory responses (31, 32).

Prolonged effects of CHBP and underling mechanisms are worth exploring. In this study, therefore, the effect of CHBP on IR and/or CsA-induced combinational injuries in mouse kidneys at 2 and 8 weeks (to avoid prolonged time increasing mortality) and kidney epithelial cells, were further investigated. We demonstrated that CHBP protected kidneys against transplant-related injuries, which might be attributed to different mechanistic signaling pathways at two time points, but both with reduced CASP-3. CHBP and CsA may share comparable immune regulations with the reduction of apoptosis in IR kidneys, whereas CHBP and/or CASP-3siRNA protected kidney epithelial cells against CsA-induced apoptosis.

## Materials and Methods

### Renal IR Injury Model

**Figure 1:**
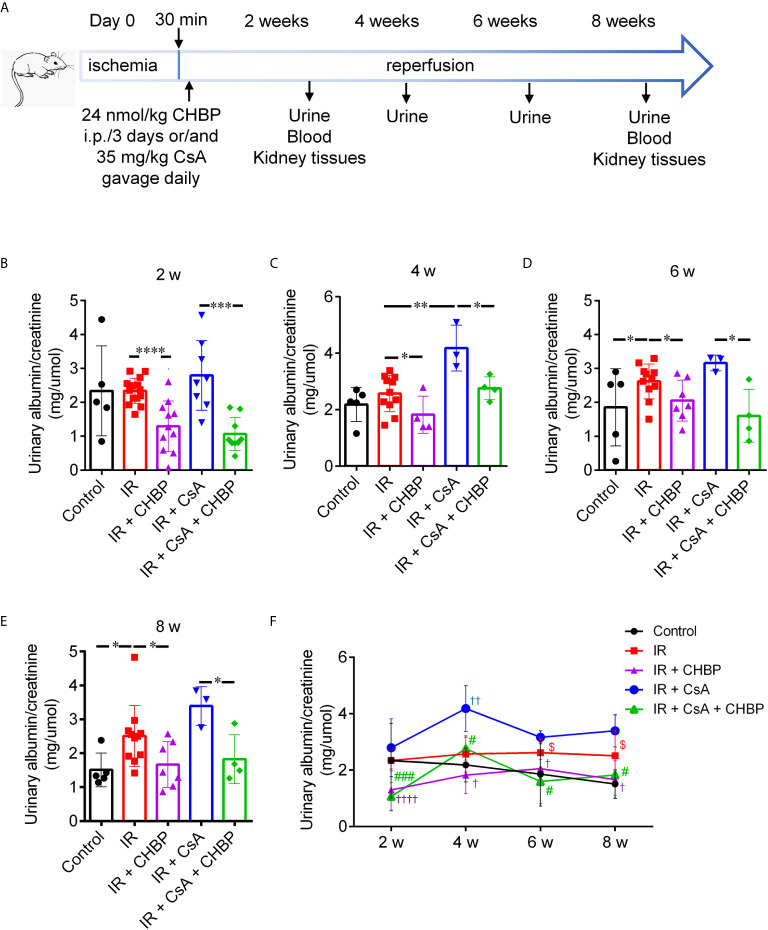
Schematic picture of *in vivo* study design and urinary albumin/creatinine. **(A)** Bilateral renal pedicles were clamped for 30 min followed by reperfusion for 2 and 8 weeks. Approximately 35 mg/kg CsA gavage daily, and/or 24 nmol/kg CHBP intraperitoneal injection every 3 days. Urine samples were collected every 2 weeks, blood samples were drawn and animals were sacrificed at 2 and 8 weeks. **(B–E)** The ratio of urinary albumin/creatinine was shown in the IR group with/without CsA treatment at 2, 4, 6 and 8 weeks. *P ≤ 0.05, **P ≤ 0.01, ***P ≤ 0.001, ****P ≤ 0.0001. **(F)** The dynamic profile of urinary albumin/creatinine at all-time points. ^†^ *P < *0.05, ^††^ *P* ≤ 0.01, ^††††^ *P* ≤ 0.0001 versus IR; ^#^ *P < *0.05, ^###^ *P* ≤ 0.0005 versus IR + CsA and ^$^ *P < *0.05 versus the control. Data were expressed as the mean ± SD of each group (n ≥ 3).

Male BALB/c mice 25–30 g were randomly divided into five groups: Control, IR, IR + CsA, IR + CHBP, and IR + CsA + CHBP, with 5–8 mice in each group using power calculation according to the key parameters of our previous studies (19–21). All animal procedures were performed according to the guidelines from the Animal Care and Use Committee of Nantong University and the Animal Care Ethics Committee of Jiangsu Province. Mice were anesthetized by 1% pentobarbital (0.01 ml/g), and bilateral renal pedicles were clamped for 30 min and reperfused for 2 or 8 weeks. The color changing of kidneys was confirmed for the efficacy of occlusion and reperfusion. For the sham control, abdominal cavity and renal pedicels were exposed without occlusion. CsA (Novartis Pharma GmbH, Eberbach, Germany) was dissolved in olive oil, gavage daily, 35 mg/kg body weight (BW). CHBP (Shanghai Institute of Materia Medica, Chinese Academy of Sciences) was dissolved in 0.9% saline, intraperitoneally injected after reperfusion every 3 days, 24 nmol/kg BW (19, 27). Urine samples were collected every 2 weeks using metabolic cages and used all 2-week urine samples including end time point collection at 2 weeks end time points for analysis. At 2 and 8 weeks, the blood was drawn, animals were sacrificed and harvested kidneys were fixed with 10% (w/v) neutral buffered formalin or snap frozen in liquid nitrogen (**Figure 1A**). However, not all mice survived to the end point, but at least n ≥3 were used for the each group in all detections. Urinary albumin/creatinine and serum creatinine (SCr) were measured using an automatic biochemistry analyzer (Siemens, Berlin, Germany).

### TCMK-1 Cell Model

TCMK-1 cells (a mouse kidney epithelial cell line, CCL­139™, American Type Culture Collection, Manassas, USA) (18) were incubated in DMEM/F12 medium supplemented with 10% (v/v) of fetal bovine serum (FBS, Gibco Technologies, Logan, USA), 100 unit/ml penicillin G (Gibco), and 100 μg/ml streptomycin (Gibco) at 37°C in 5% CO_2_ atmosphere. The cells grown to about 80% confluent in monolayer in 6-well plates were treated by CsA (2.5, 5, 10, 20 and 40 μg/ml) with/without CHBP (2.5, 5, 10, 20 and 40 ng/ml) for 24 h. TCMK-1 cells were transfected with CASP-3siRNA (10, 20, 30 and 40 nM) by Lipofectamine^@^ RNAiMAX 4–6 h before above treatments (18, 26). The sequences of CASP-3siRNA are: 5’-GCUUCUUCAGAGGCGACUAtt-3’ and 5’-UAGUCGCCUCUGAAGAAGCta-3’, with the negative control siRNA not targeting any known mammalian genes (Thermo Fisher Scientific, Waltham, USA). Obtained data were from three independent experiments at least (**Figure 7A**).

### Histological Assessment

Approximately 4 µm paraffin sections were stained by hematoxylin and eosin (H&E). The tubulointerstitial damage (TID) include tubular dilation and vacuolation, interstitial expansion (edema or fibrosis), inflammatory cell infiltration, protein or cell casts in tubular lumina was semi-quantitatively evaluated using 0–4 scales: <5% score 0; 5–25% score 1; 25–50% score 2; 50–75% score 3; and exceeding 75% score 4. Approximately 12 cortical fields of each section at 200× magnification were scored by two examiners blinded to experimental groups (19, 22, 27).

Tubulointerstitial fibrosis was evaluated by Masson’s trichrome staining of collagen deposition (19). Twenty fields of cortical areas were quantified at 400× magnification using the Image-Pro Plus software (Media Cybernetics, Rockville, USA).

### Detection of Apoptotic Cells

Fragmented DNAs in paraffin sections were *in situ* end labeled (ISEL) using an ApopTag peroxidase kit (S7100, Appligene Oncor, Illkirch, France) and 3’-amino-9-ethylcarbazole (SK-4200, AEC, Vector, Burlingame, USA) as substrate. Apoptotic cells were counted in tubular, tubular luminal and interstitial areas in 20 fields at 400× magnification (19, 22).

TCMK-1 cells were tripsinized, re-suspended in binding buffer and incubated with FITC-conjugated annexin-V and propidium iodide (PI) for 15 min. Approximately 10,000 cells were analyzed by BD FACS Calibur flow cytometry (BD Biosciences, Franklin Lakes, USA). Living cells (Annexin-V−/PI−), early apoptosis (Annexin-V+/PI−), later apoptosis (Annexin-V+/PI+) or necrosis (Annexin-V−/PI+) were shown as quadrant dot plots and the percentage of the gated cells (18, 26).

### Immunohistochemistry

Immunohistochemistry staining of F4/80, a marker of macrophages, was performed on 4 µm paraffin sections treated by 40 µg/ml proteinase K (Sigma, Dorset, UK) at 37°C, 30 min for antigen retrieving. F4/80 (1:50, ab11101, Abcam, Cambridge, UK) was incubated at 4°C overnight. DAKO EnVision™ + Dual Link System-HRP was used as the secondary antibody (DAKO, Glostrup, Denmark). The antibody binding was revealed by 3,3’-diaminobenzidine (SK-4100, DAB, Vector, Burlingame, USA) with hematoxylin counterstaining. The number of macrophages in the tubular, interstitial, and tubular lumen areas were semi-quantitatively analysed in 20 fields at 400× magnification.

### Western Blotting Analysis

Kidney proteins (25 µg) were separated by electrophoresis using 12–15% polyacrylamide gels and blotted onto 0.4 μm polyvinylidene difluoride membranes (Roche Diagnostics GmbH, Mannheim, Germany). Membranes were blocked with 5% milk before incubated with full-length CASP-3 (1:200, sc-7148, Santa Cruz Biochemicals, Santa Cruz, USA), HMGB1 (1:1,000, #3935, Cell Signaling Technology, Beverly, USA) or β-actin antibody (1:1,000, ab6276, Abcam, Cambridge, UK). After incubation with peroxidase-conjugated secondary antibody, the binding was revealed by ECL substrate using a Molecular Imager Chemi Doc XRS+ system and semi-quantitatively analyzed by Image Lab Software (Bio-Rad, Berkeley, USA). Optical volume density values of target proteins were expressed as the percentage of average controls after loading correction by β-actin (19, 22).

### RT-qPCR

The level of CASP-3 mRNA in kidneys and TCMK-1 cells was detected by reverse transcription (RT) quantitative real-time polymerase chain reaction (qPCR) using a StepOne Plus Real-Time PCR system (Applied Biosystems, Foster City, USA). Approximately 1 μg of total extracted RNAs was used for RT of complementary DNA (cDNA). cDNA was amplified in qPCR reaction buffers (15, 18, 21, 22). The probes of CASP-3 and glyceraldehyde-3-phosphate dehydrogenase (GAPDH) were 6-carboxy-fluorescein (FAM) labeled (Thermo Fisher Scientific). The reaction conditions were as follows: 2 min at 95°C, 40 cycles of 95°C for 10s, then 60°C for 10s. CASP-3 mRNA normalized by GAPDH was calculated against control kidneys using a 2^−ΔΔ^ *^C^* ^t^ method.

### Antibody Array Analysis

Eighteen well-characterized phosphorylated or cleaved signaling molecules were simultaneously detected using 50 µg kidney proteins by a slide-based PathScan^®^ Intracellular Signaling Array Kit (#7323, Cell Signaling Technology) according to the manufacturer’s instructions. Images were acquired by briefly exposing the slide to the Chemi Doc XRS+ system (Bio-Rad), and semi-quantitatively analyzed by scanning volume density using Alpha View Software 3.3 (Cell Biosciences, Santa Clara, USA) (33).

### Statistical Analysis

Data were represented as the mean ± standard deviation (SD). One-way analysis of variance (ANOVA) was used to compare more than two groups, while two-tailed Student’s *t*-test was used for two groups using GraphPad Prism 6.0 (GraphPad Software, San Diego, USA). *P* ≤0.05 was considered to be statistically significant.

## Results

### CHBP Reduced Urinary Albumin/Creatinine

The ratio of urinary albumin to creatinine (mg/µmol) was increased by CsA compared with that produced by IR kidneys at 4 weeks (**Figures 1B, C**), while this ratio was also increased by IR compared with the sham controls at 6 and 8 weeks (**Figures 1D, E**). Most impressively, CHBP significantly decreased urinary albumin/creatinine produced by IR kidneys with/without CsA treatment at all-time points (**Figures 1B–E**).

The dynamic profile of urinary albumin/creatinine was higher in the IR group compared with the control and CHBP treated groups, and further increased by CsA at all-time points over 2–8 weeks (**Figure 1F** **)**. The difference between the control and IR groups was enlarged by prolonged time, and minimized by CHBP treatment with/without CsA. The change trends in two CsA groups were surprisingly similar, peaked at 4 weeks, decreased at 6 weeks and leveled up to 8 weeks.

Nevertheless, there was no significant difference in the level of SCr between all groups at 2 and 8 weeks (**Supplementary Figures 1A, B**).

### CHBP Improved Tissue Damage

**Figure 2:**
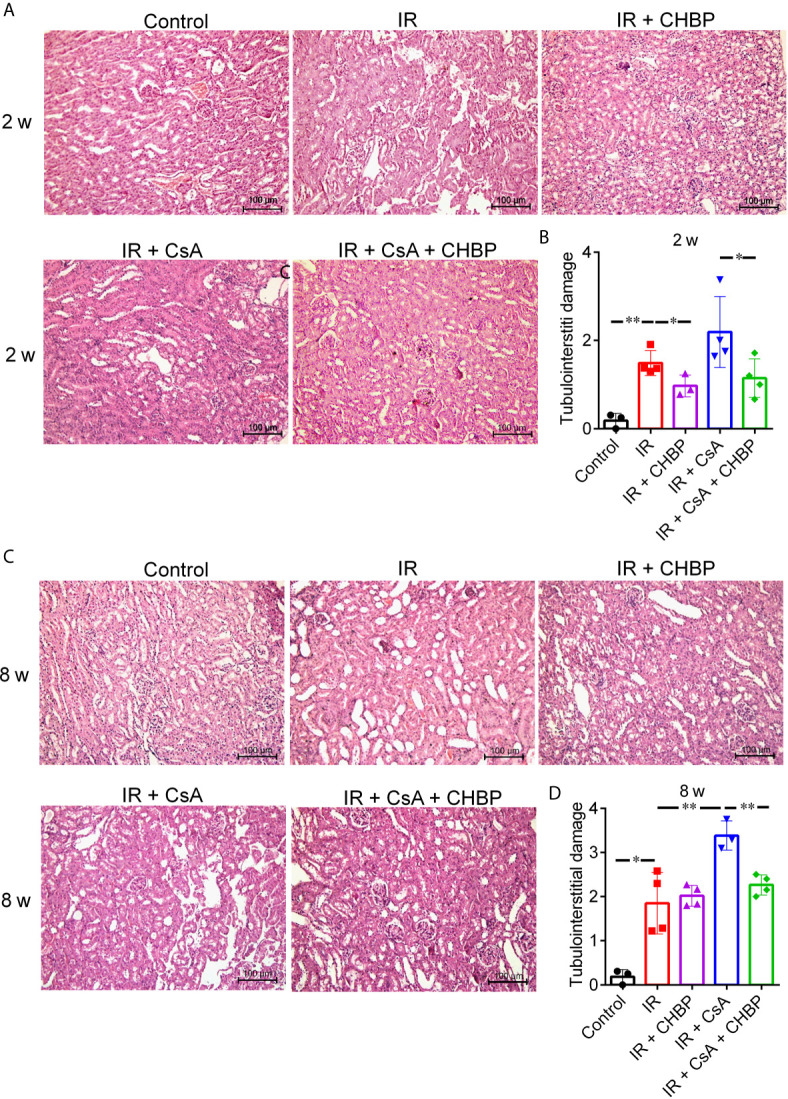
The semi-quantitative score of TID in H&E staining sections. **(A, C)** Tubular dilation, epithelial cell vacuolation, interstitial expansion with edema or inflammation, and cells or cell debris in tubular lumens were mainly seen in IR kidneys. **(B)** CHBP treatment improved the TID compared with IR and IR + CsA groups at 2 weeks. **(D)** The score was further increased in the IR + CsA group but reversed by CHBP at 8 weeks. Data were expressed as the mean ± SD of each group (n ≥ 3). *P ≤ 0.05; **P ≤ 0.01.

The semi-quantitative score of TID in H&E sections was significantly increased by IR compared with the control at both time points (**Figures 2A–D**), further increased by CsA with statistical significance at 8 weeks. Most interestingly, TID was improved by CHBP in IR kidneys at 2 weeks only, and in the CsA-treated kidneys at 2 and 8 weeks.

**Figure 3:**
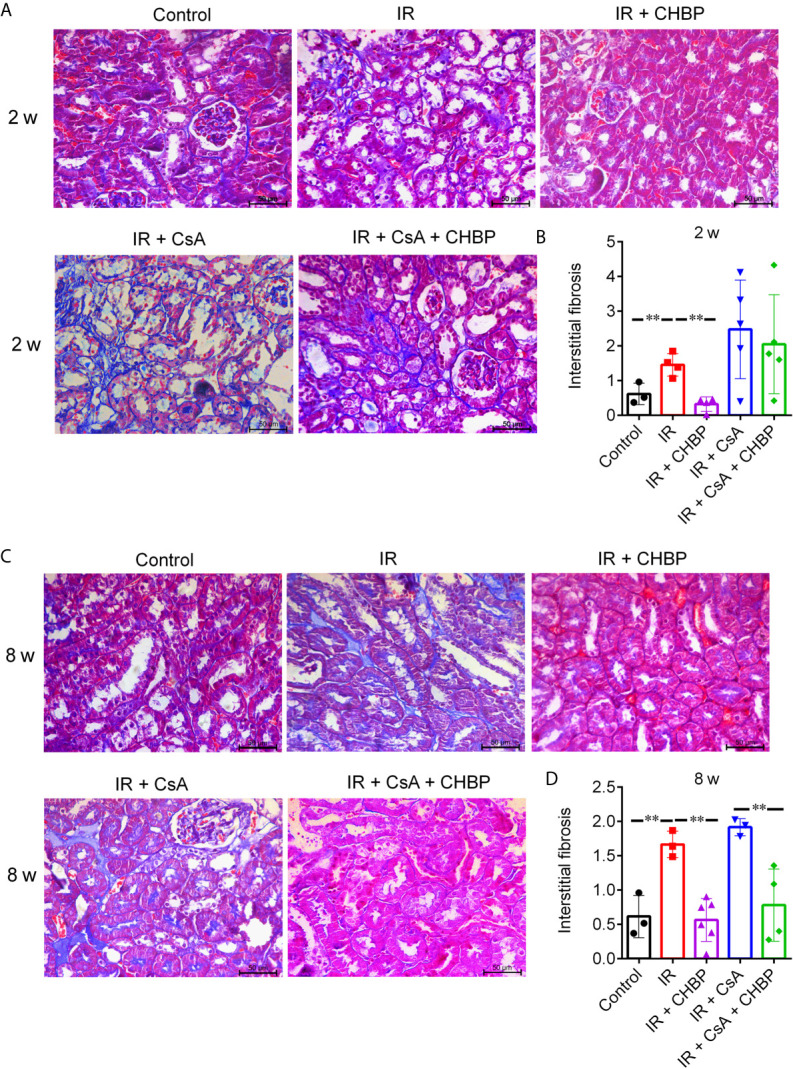
The score of interstitial fibrosis in Masson’s trichrome stained sections. **(A, C)** Masson’s trichrome staining revealed the interstitial fibrosis. **(B, D)** Interstitial fibrosis was increased by IR compared with the control, but improved by CHBP at both time points. **(D)** The score of fibrosis was significantly decreased by CHBP in the CsA treated group at 8 weeks. Data were expressed as the mean ± SD of each group (n ≥ 3). **P ≤ 0.01.

Interstitial fibrosis revealed in Masson’s trichrome stained sections was significantly increased by IR compared with the control, but improved by CHBP at both time points (**Figures 3A–D**), while CsA increased interstitial fibrosis was significantly decreased by CHBP at 8 weeks only.

### Cellular Apoptosis in Kidneys Reduced by CHBP and CsA

**Figure 4:**
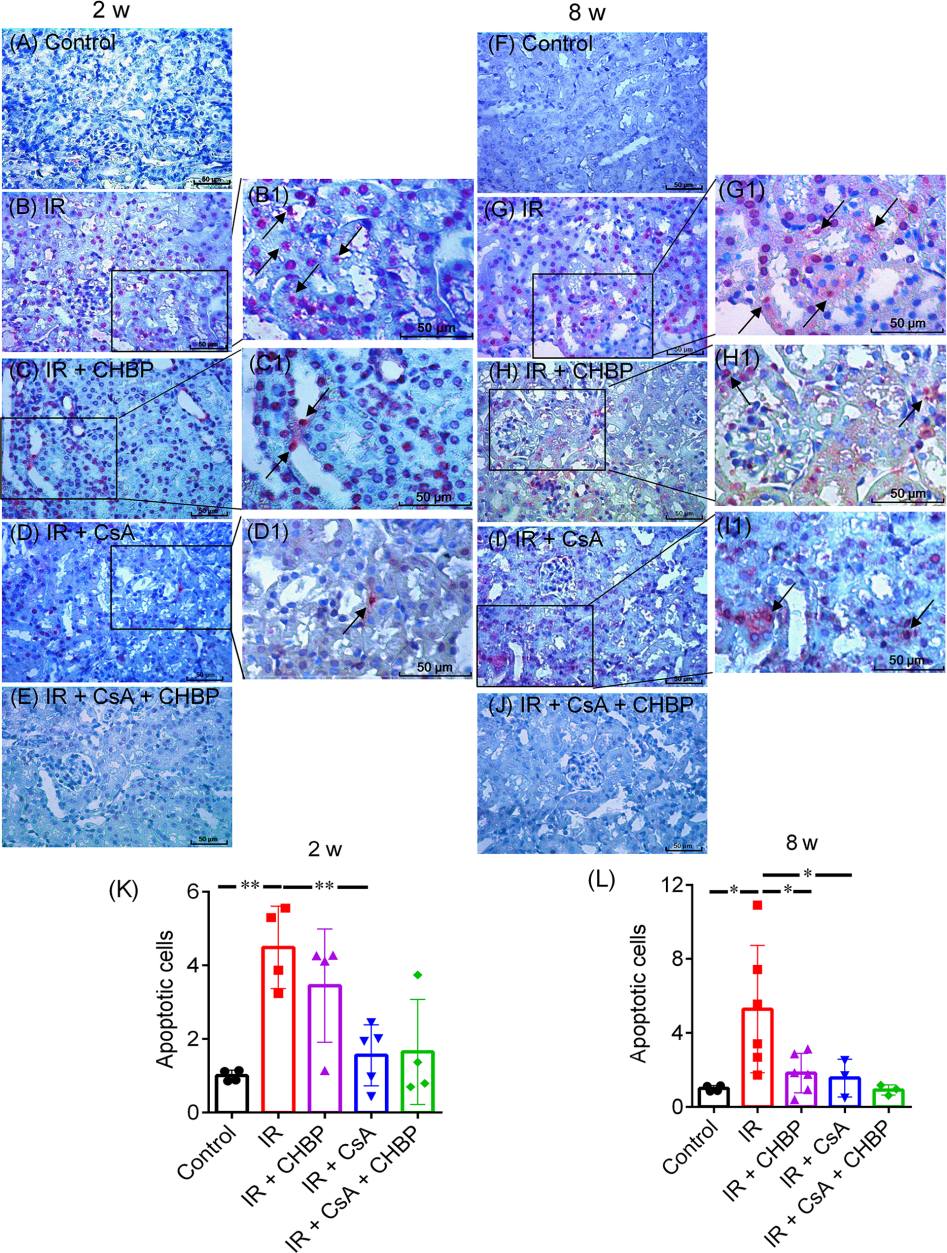
Apoptotic cells detected by labelling fragmented DNAs. **(A–J)** Apoptosis were mainly shown in tubular and interstitial areas. **(B1–D1, G1–I1)** Enlarged pictures were from **(B–D)** and **(G–I)**. **(K, L)** The number of apoptotic cells was greatly increased by IR, but decreased by CsA at 2 and 8 weeks. CHBP treatment decreased apoptotic cells in the IR kidneys at 8 weeks. Data were expressed as the mean ± SD of each group (n ≥ 3). *P ≤ 0.05; **P ≤ 0.01.

Apoptotic cells detected by ISEL were mainly located in the tubular and interstitial areas (**Figures 4A–J**), some of them had polymorphic nuclei (4B1, C1), with very few seen in glomerular areas. The total number of apoptotic cells, summed up from that in tubular and interstitial areas and tubular lumina, was greatly increased by IR, but decreased by CsA at 2 and 8 weeks, and by CHBP at 8 weeks (**Figures 4K, L**).

### Macrophages in Kidneys Affected by CHBP and CsA

F4/80+ macrophages were mainly located in the interstitial areas (**Supplementary Figure 2A**). The number of macrophages in interstitial areas increased by IR was numerically decreased by CsA and CHBP at 2 weeks, but did not reach statistical significance (**Supplementary Figure 2B**). In addition, the increase trend induced by CsA was also seen at 8 weeks, but there was no statistical significance (**Supplementary Figure 2C**).

### CASP-3 mRNA and Its Active Protein Reduced by CHBP

**Figure 5:**
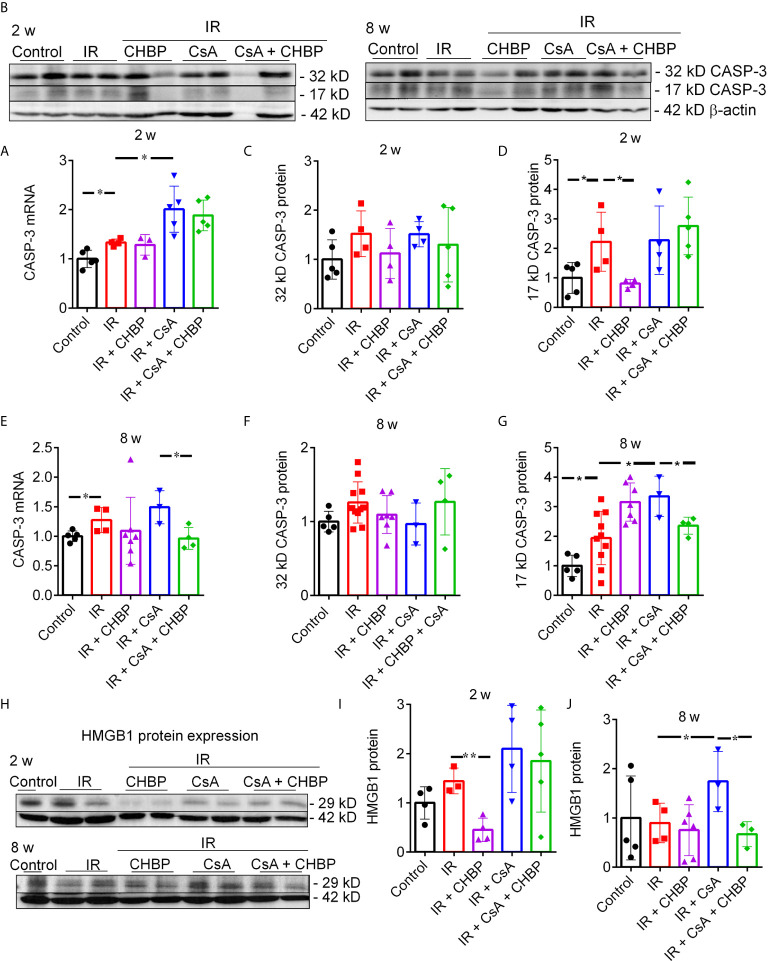
*CASP-3* mRNA and protein, and HMGB1 protein expression. **(A, E)** The relative expression of *caspase 3* mRNA was increased by IR at both time points, and further increased by CsA at 2 weeks, but decreased by CHBP in the CsA group at 8 weeks. **(B–D, F, G)** The level of 17 kD active CASP-3 protein was increased by IR and reversed by CHBP at 2 weeks, and increased by CsA at 8 weeks and also reversed by CHBP. **(H–J)** The expression of HMGB1 protein in 2 and 8 weeks was detected by western blotting. The level of HMGB1 was significantly decreased by CHBP in the IR group at 2 weeks, increased by CsA compared with the IR group and reversed by CHBP at 8 weeks. Data were expressed as the mean ± SD (n ≥ 3). The volume density of western blots was corrected by against 42 kD β-actin as a loading control. *P ≤ 0.05; **P ≤ 0.01.

CASP-3 mRNA measured by qPCR was increased by IR at both time points, and further increased by CsA at 2 weeks, but decreased by CHBP in the CsA group at 8 weeks (**Figures 5A, E**).

CASP-3 protein was detected by western blotting, (**Figures 5B, C, F**). Approximately 17 kD active CASP-3 was increased by IR and reversed by CHBP at 2 weeks; further increased by CsA at 8 weeks was also reversed by CHBP (**Figures 5D, G**).

### HMGB1 Protein Decreased by CHBP

CHBP decreased the increased HMGB1 protein by IR at 2 weeks (**Figures 5H, I**) and the further increased HMGB1 by CsA compared with IR kidneys at 8 weeks (**Figures 5H, J**), detected by western blotting.

### Different Proteins Changed at 2 and 8 Weeks

**Figure 6:**
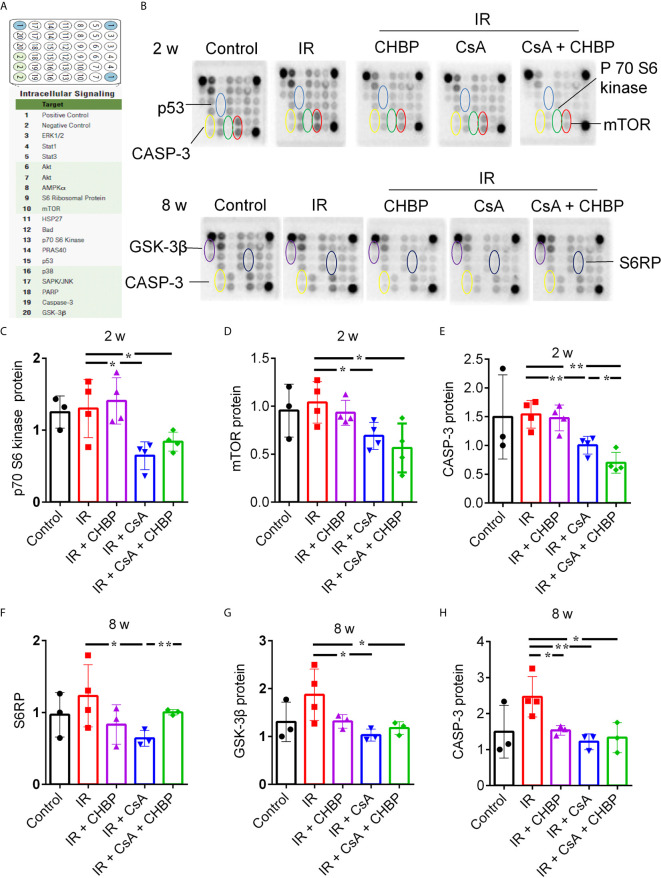
Simultaneous detection of 18 proteins by slide-based antibody array. **(A, B)** The expression of 18 proteins in the kidneys revealed by slide-based antibody array. **(C–E)** CsA decreased p70 S6 kinase, mTOR and caspase-3 at 2 weeks. **(F–H)** S6RP was decreased by CsA in IR kidneys, but reversed by CHBP. CsA decreased GSK-3β and caspase-3 at 8 weeks in IR kidneys with/without CHBP treatment. **(E–H)** Caspase-3 was decreased by CHBP in CsA treated IR kidneys at 2 weeks and in IR kidneys at 8 weeks. Data were expressed as the mean ± SD (n ≥ 3). The volume density was corrected by against the control. *P ≤  0.05; **P ≤ 0.01.

Eighteen proteins in the kidney were detected at the same time by slide-based antibody array (**Figures 6A, B**). In IR kidneys with/without CHBP treatment, CsA decreased p70 S6 kinase, mTOR and CASP-3 at 2 weeks, and GSK-3β and CASP-3 at 8 weeks (**Figures 6C–E, G, H**). However, S6 ribosomal protein (S6RP) was decreased by CsA in IR kidneys, but reversed by CHBP (**Figure 6F**). CASP-3 was decreased by CHBP in CsA treated IR kidneys at 2 weeks and in IR kidneys at 8 weeks (**Figures 6E–H**).

### CHBP Reduced CASP-3 and Apoptosis Increased by CsA in TCMK-1 Cells

**Figure 7:**
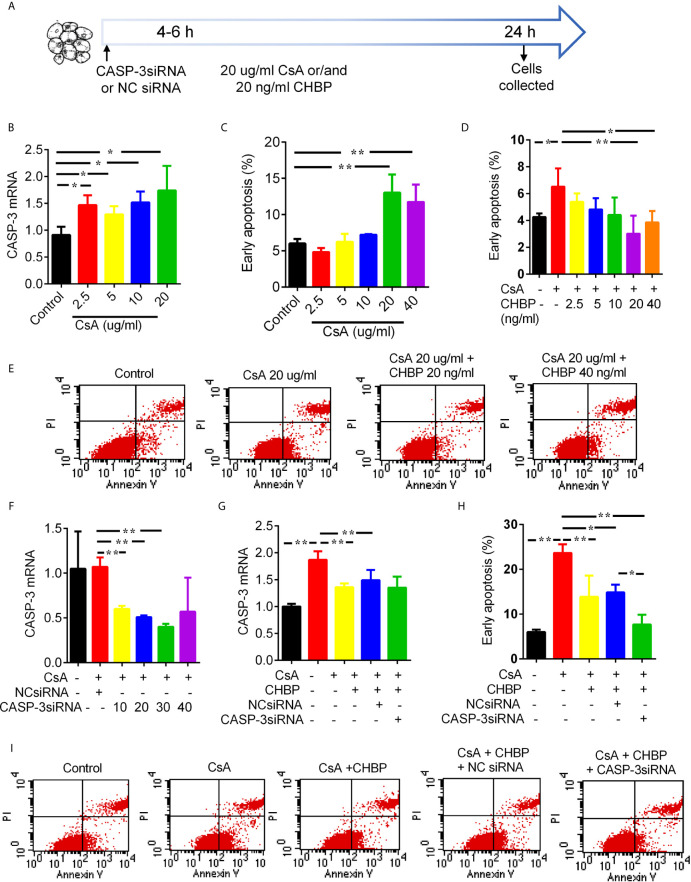
Schematic picture of *in vitro* study design and effects of CsA, CHBP and CASP-3siRNA on TCMK-1 cells. **(A)** TCMK-1 cells were transfected with CASP-3siRNA 4–6 h before treated by CsA with/without CHBP for 24 h. **(B)** The level of CASP-3 mRNA was gradually increased by 2.5–20 μg/ml CsA. **(C)** The percentage of early apoptotic cells was significantly increased by CsA by 20–40 μg/ml and peaked at 20 μg/ml. **(D, E)** Early apoptotic cells was gradually reduced by 2.5–40 ng/ml CHBP, and reached statistical significance at 20–40 ng/ml. **(F)** The expression of CASP-3 mRNA was reduced by 10–30 nM/ml CASP-3siRNA compared with the control treated by 20 μg/ml CsA or with NCsiRNA, but the suppression was maximized at 30 nM/ml with 64.85% reduction. **(G)** CASP-3 mRNA were significantly increased by 20 μg/ml CsA, but reversed by 20 ng/ml CHBP with/without 30 nM CASP-3siRNA. **(H, I)** Early apoptotic cells was significantly increased by 20 μg/ml CsA, but reversed by CHBP with/without CASP-3siRNA or NCsiRNA. Data were expressed as the mean ± SD, (n = 3). *P < 0.05, **P < 0.01.

CASP-3 mRNA was gradually increased by 2.5–20 μg/ml CsA (**Figure 7B**), so was early apoptotic cells, and reached statistical significance at 10 and 20 µg/ml (**Figure 7C**). However, early apoptotic cells were gradually reduced by 2.5–40 ng/ml CHBP, and reached statistical significance at 20–40 ng/ml (**Figures 7D, E**).

### CASP-3siRNA Decreased CASP-3 mRNA and Apoptosis in TCMK-1 Cells

CASP-3 mRNA was reduced by 10–30 nM/ml CASP-3siRNA compared with the control treated by 20 μg/ml CsA or with NCsiRNA. The suppression was maximized by CASP-3siRNA at 30 nM/ml with 64.85% reduction (**Figure 7F**). CASP-3 mRNA were significantly increased by 20 μg/ml CsA, but reversed by 20 ng/ml CHBP with/without 30 nM CASP-3siRNA (**Figure 7G**).

Early apoptotic cells was also significantly increased by 20 μg/ml CsA, but reversed by CHBP with/without CASP-3siRNA or NCsiRNA (**Figures 7H, I**). Most interestingly, the combined treatment of CHBP and CASP-3siRNA further reduced early apoptotic cells compared with the CsA + CHBP + NCsiRNA group.

## Discussion

The renoprotection of CHBP against IR injury and HBSP against IR and CsA-induced injury has been shown previously (18, 20, 26, 27). However, there has been no report regarding the long-term effect of CHBP on IR and CsA-induced combinational injury in mouse models. In this study, the renoprotection of CHBP was confirmed not only in renal IR injury but also with CsA nephrotoxicity at 2 and 8 weeks, which better mimicked human kidney transplantation. Urinary albumin/creatinine was more sensitive than SCr in detecting transplant-related injuries, which was supported by changes in apoptosis, inflammation, TID and tubulointerstitial fibrosis. The renoprotective mechanisms of CHBP on IR injury and CsA nephrotoxicity might be different, with decreased mTOR and p70 S6 kinase at 2 weeks, GSK-3β and S6RP at 8 weeks, and CASP-3 protein at both time points. These changes may reflect the dynamic nature of two different injuries, in terms of IR injury gradually eased off in 2 weeks, but CsA nephrotoxicity continuously built up over 8 weeks. In addition, the effect of CsA on apoptosis appeared differently in kidneys having different cell types and kidney epithelial cells *in vitro* (**Supplementary Figure 3**).

CsA is widely used in organ transplantation to prevent rejection, but its nephrotoxicity cannot be ignored (34, 35). Previous long-term studies of kidney IR injury revealed that CsA increased interstitial inflammation and renal fibrosis (19, 21). To explore the pathogenesis of CAD in-depth, the mouse model subjected to renal transplant-related injuries, IR injury and CsA nephrotoxity, was established at 2 and 8 weeks. Urinary albumin/creatinine was increased by IR injury, together with TID, interstitial fibrosis and apoptotic cells in tubular areas. CsA further increased urinary albumin/creatinine from 2 and 4 weeks. However, SCr was not statistically different between groups. Urinary albumin/creatinine, therefore, is a non-invasive and more sensitive biomarker than SCr in monitoring renal transplant-related injuries for middle/long-term.

CsA reduced apoptotic cells in IR kidneys at both time points, which was contradictory to the change in TID, CASP-3, HMGB1, tubulointerstitial fibrosis, and CsA-induced early apoptosis in TCMK-1 cells. Apoptosis is an inevitable phenomenon in transplant-related kidney damage (36), while the apoptosis in different types of cells could lead to inverse outcomes: on one hand excessive TEC apoptosis resulting in tubular atrophy and dysfunction; on the other hand, inflammatory cell apoptosis facilitating the clearance of inflammation, renal structure remodeling and functional recovery (37, 38). Furthermore, the number of macrophages was detected to evaluate the effect of CHBP/CsA on infiltrated inflammatory cells. The results showed that IR increased macrophages mainly located in the interstitial areas were numerously decreased by CsA and further decreased by CHBP at 2 weeks, both of which were reversed at 8 weeks. Because of the large variation between individual animals, there was no any statistically significant difference between groups. Therefore, we concluded that CsA might help renal structure remodeling and functional recovery by decreased apoptosis of TECs at 2 weeks. However, the intensive inflammation at 8 weeks, with decreased apoptosis of inflammatory cells, might aggravate the structural damage of kidneys, impede functional recovery, and lead to tubulointerstitial fibrosis that characterizes CsA-induced long-term nephrotoxicity (21).

The renoprotection of CHBP against IR injury and/or CsA nephrotoxicity was also demonstrated. Given that urinary albumin/creatinine was decreased by CHBP in IR injury and/or CsA groups from 2, 4, 6 to 8 weeks, and TID, active CASP-3 and HMGB1 expression and fibrosis were improved by CHBP in IR kidneys at 2 weeks. IR-induced apoptotic cells were decreased by CHBP at 8 weeks only, which implies the nature of the model in terms of the apoptosis in different types of cells. In addition, CHBP improved interstitial fibrosis, decreased CASP-3 mRNA and 17 kD protein, and HMGB1 in IR kidneys with additional CsA only at 8 weeks, which suggests that the accumulated nephrotoxicity of CsA might further facilitate the renoprotection of CHBP. Therefore, the renoprotection of CHBP was embodied in either improving IR injury, TID and inflammation at 2 weeks, or reducing accumulated CsA nephrotoxicity, apoptosis and interstitial fibrosis at 8 weeks.

To further investigate whether CHBP has comparable immune regulating responses with CsA or what different underlying mechanisms involved in IR injury with/without CsA at 2 and 8 weeks, the expression of multiple mechanistic signaling proteins was analyzed in the same sample by a slide based antibody array. p70 S6 kinase, mTOR, and CASP-3 were decreased by CsA in IR kidneys at 2 weeks, while S6RP, GSK-3β and CASP-3 were reduced at 8 weeks. CHBP also downregulated CASP-3 in IR + CsA treated kidneys at 2 weeks and in IR kidneys at 8 weeks, but upregulated S6RP at 8 weeks in IR kidneys with CsA treatment. It suggests that CHBP protecting IR injury and/or CsA nephrotoxicity might be associated with different mechanistic signaling pathways, with CASP-3 at convergent point. Therefore, CASP3siRNA therapy might be an additional treatment to CHBP for transplant-related renal injury.

Our previous studies have shown the up-regulated CASP-3 in TECs associated with apoptosis and inflammation in different IR-induced acute and chronic kidney injuries (3, 18, 19, 21, 39), and the renoprotection of CASP-3siRNA (22, 40, 41). In order to dissect the effect and mechanism of CHBP and CsA, TCMK-1 cells were stimulated by CsA, and also treated with CHBP and/or CASP-3siRNA. Approximately 20 μg/ml CsA and 20 ng/ml CHBP were most effective at 24 h. CASP-3 mRNA and early apoptotic cells were dose-dependently increased by CsA, but reversed by CHBP with or without CASP-3siRNA, while early apoptotic cells were further decreased by CHBP together with CASP-3siRNA. These results reveal that CHBP combined with CASP-3siRNA had a profound protection against CsA-induced TEC injury by anti-apoptosis. The synergistic effect of both was also revealed in mouse IR kidneys at 48 h in one of our more recent studies (42).

This finding needs to be further validated in animal models at the suitable stage of injury using modified CASP-3siRNA *via* optimized delivery route. The results from this study guide our near future investigations: whether CHBP has comparable immune regulating function with CsA; how different is the underlying mechanism of IR injury and/or CsA nephrotoxity at 2 and 8 weeks; how to combine CHBP with siRNA for therapeutic applications at different stages of injury. The precise mechanism of CHBP and combined effect with CASP-3siRNA are worth further investigating.

In conclusion, the predominant renoprotection of CHBP against IR injury was at 2 weeks, and against CsA nephrotoxicity was at 8 weeks, with urinary albumin/creatinine as a good biomarker in monitoring the progression of transplant-related chronic injuries. The renoprotection of CHBP was associated with different mechanistic signaling proteins such as mTOR at 2 weeks and GSK-3β at 8 weeks, and CASP-3 at both time points. CHBP combined with CASP-3siRNA had a profound protection against CsA-induced injury in TECs, although CsA divergently affected apoptosis in kidneys and TECs.

## Data Availability Statement

The raw data supporting the conclusions of this article will be made available by the authors, without undue reservation.

## Ethics Statement

The animal study was reviewed and approved by Animal Care and Use Committee of Nantong University and the Jiangsu Province Animal Care Ethics Committee.

## Author Contributions

YuZ, FL, and YW contributed equally to this study. BY participated in research design, paper rewriting and funding acquisition. YuZ performed experiments, data analysis and paper preparation. FL, YW, YiZ, AL, and JW participated in the performance of experiments and data analysis. CY performed surgical procedures. TZ, MN, and YF advised research design and performance. All authors contributed to the article and approved the submitted version.

## Funding

This work was supported by National Natural Science Foundation of China (Grant No. 81170689, 81570677 and 81873622 to BY).

## Conflict of Interest

The authors declare that the research was conducted in the absence of any commercial or financial relationships that could be construed as a potential conflict of interest.

## Publisher’s Note

All claims expressed in this article are solely those of the authors and do not necessarily represent those of their affiliated organizations, or those of the publisher, the editors and the reviewers. Any product that may be evaluated in this article, or claim that may be made by its manufacturer, is not guaranteed or endorsed by the publisher.
