# Supplementary material for: Long-Term Protection of CHBP Against Combinational Renal Injury Induced by Both Ischemia–Reperfusion and Cyclosporine A in Mice
Source: Front Immunol. 2021 Jul 26;12:697751. doi: 10.3389/fimmu.2021.697751 (PMC8350137; doi:10.3389/fimmu.2021.697751)
Supplement: Supplementary file 1 [file DataSheet_1.pdf]

Supplementary Material

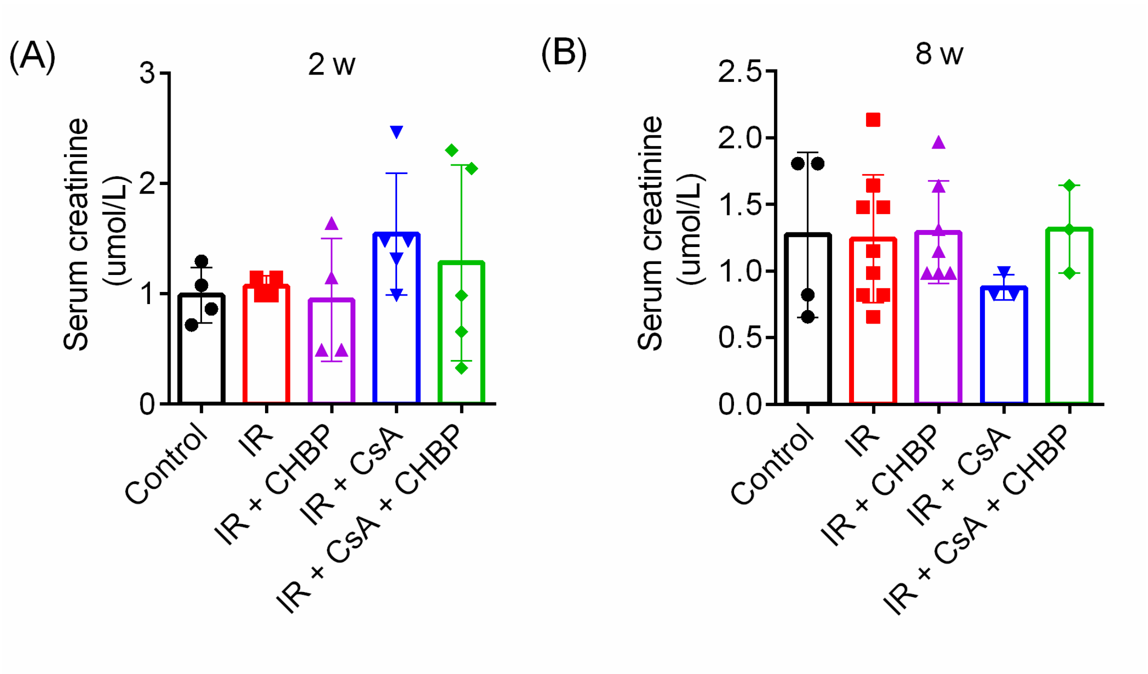

Supplementary Figure 1. The change of renal function detected by SCr.

(A, B) The level of SCr was not changed significantly by either CsA or CHBP compared with the control at 2 and 8 weeks. Data were expressed as the mean ± SD of each group (n = 6). *P < 0.05, **P < 0.01.

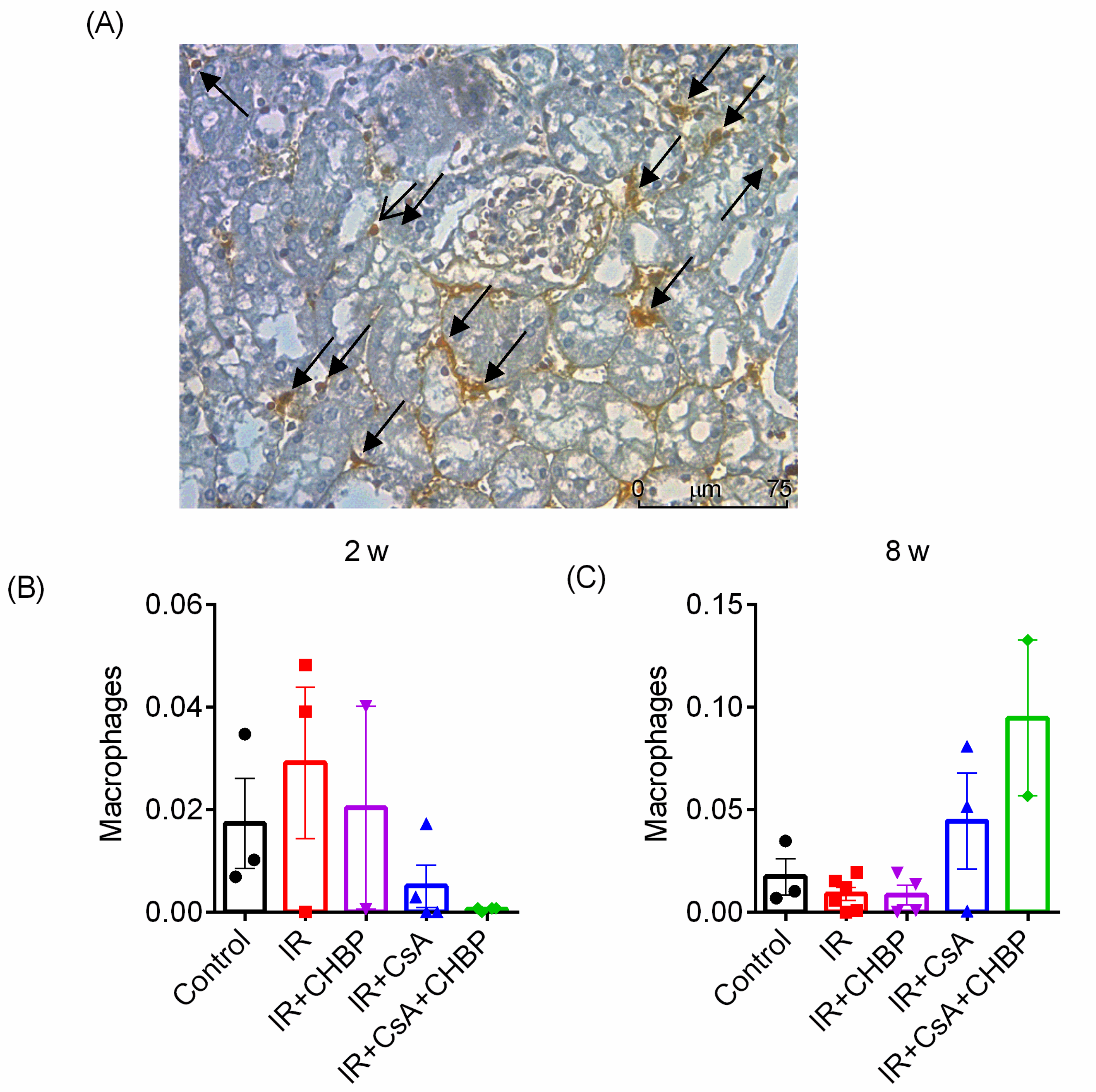

Supplementary Figure2. F4/80+ macrophages in kidneys affected by CHBP and CsA.

(A) F4/80+ macrophages indicated by arrows were mainly located in the interstitial areas. (B) The number of macrophages in interstitial was not significantly affected by CsA or CHBP at 2 weeks. (C) The number of macrophages in interstitial areas was numerically increased by CsA at 8 weeks, but no statistical significance. Data were expressed as the mean ± SD of each group (n > 3). There has no statistical significance.

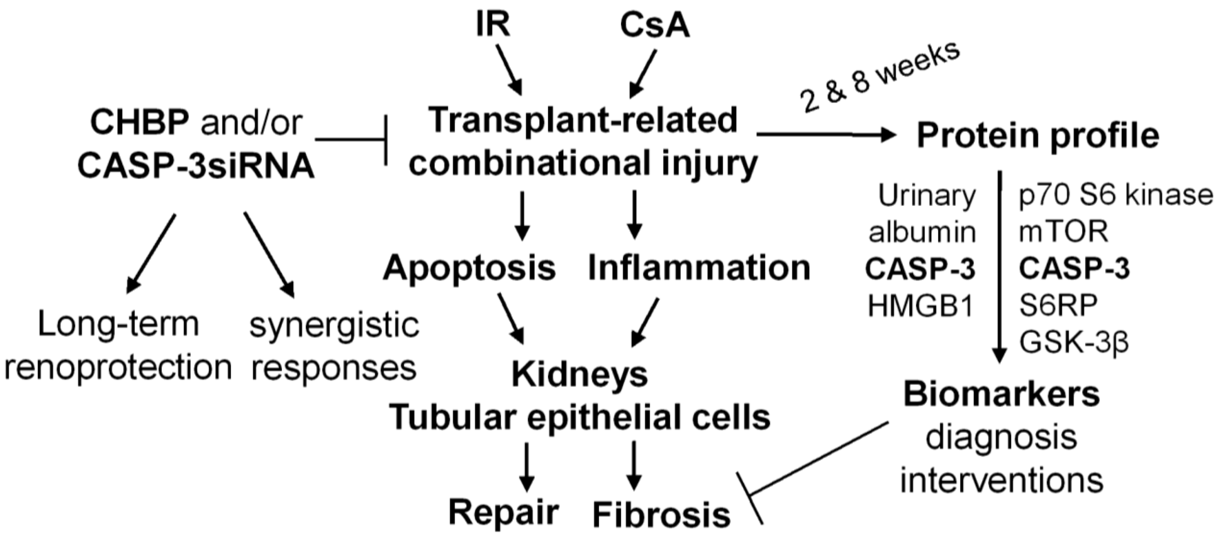

Supplementary Figure 3. The schematic illustration of the renoprotection and underlying mechanism of CHBP and/or CASP-3siRNA against IR and CsA-induced injury.

Original Data

Figure 5

(B)

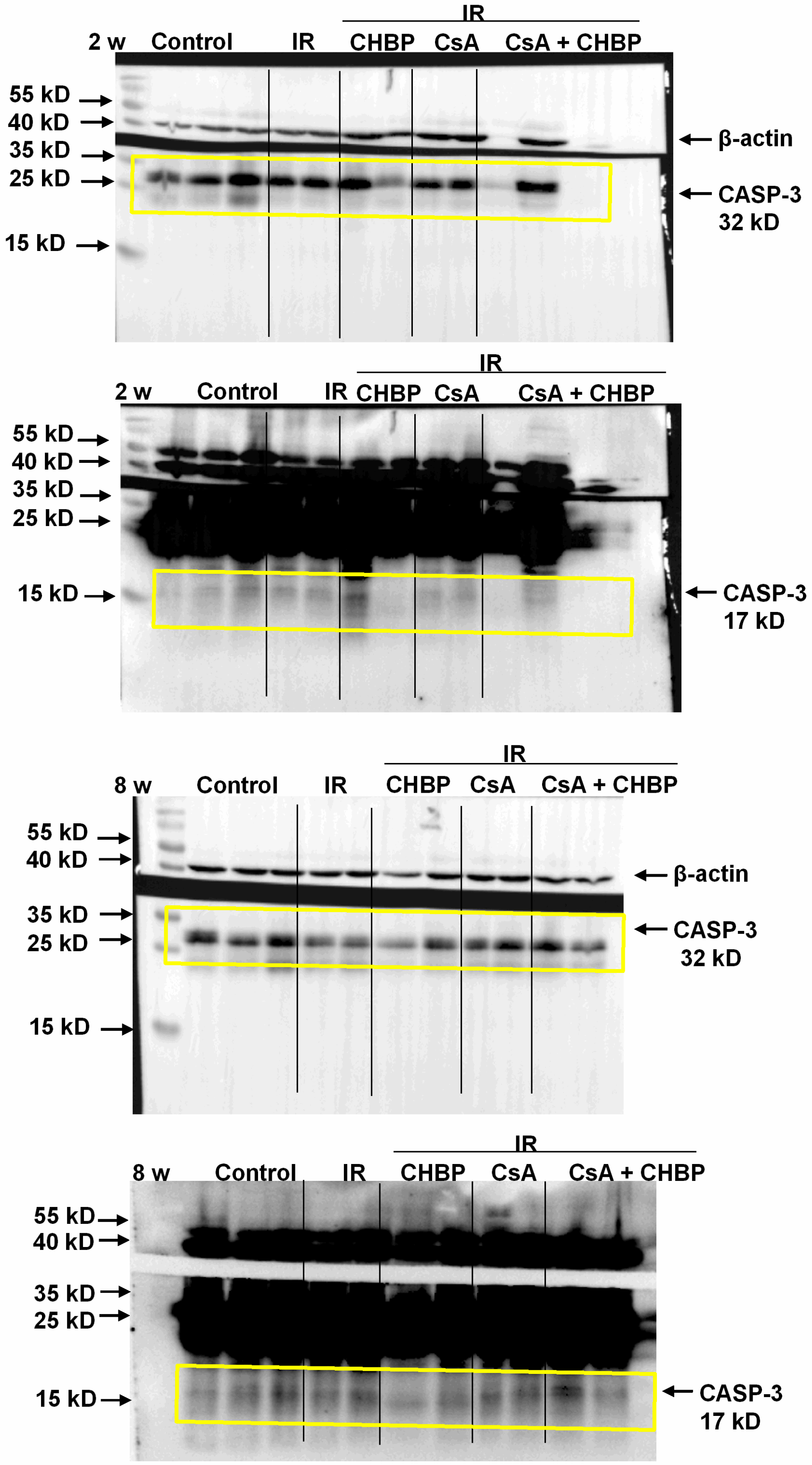

Original Data 1. The full scan of the entire original gels of Figure5 B.

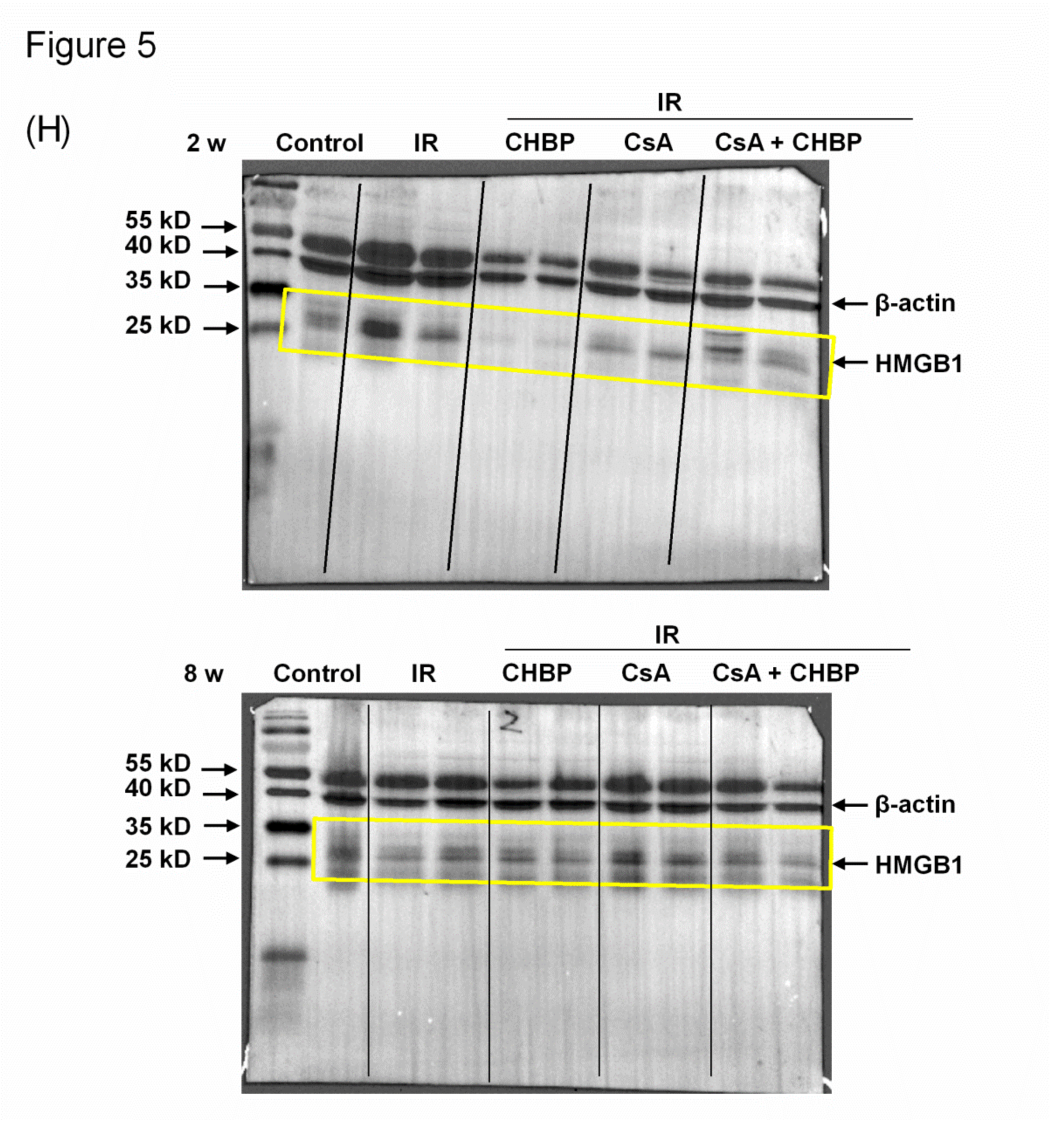

Original Data 2. The full scan of the entire original gels of Figure5 H.
